# Spatial distribution of selected coastal Sabkhas along the Southern Red Sea Coast of Egypt

**DOI:** 10.1038/s41598-025-28627-w

**Published:** 2026-03-03

**Authors:** Nada A. Younis, Galal H. El-Habaak, Hany H. El Hadek, Wael F. Galal, Mahmoud Abdel-Hakeem

**Affiliations:** 1 Geology Department Faculty of Science , Assiut University , 71516 Assiut, Egypt; 2 Department of Geology Faculty of Science , South Valley University , Qena, 83523 Egypt

**Keywords:** Sabkhas, Spatio-temporal, Remote sensing, GIS, Red Sea coast, Egypt, Climate sciences, Ecology, Ecology, Environmental sciences, Ocean sciences

## Abstract

Sabkhas are unique salt flat formations situated along the coastline and have been the subject of extensive scientific inquiry. This study delves into the formation and significance of sabkhas, along the South Red Sea coast of Egypt. Combining field observations, satellite imagery, and GIS analysis, the research unveils the processes shaping these distinct landscapes and their broader impact on the region. The study utilizes Sentinel-2 A imagery and digital elevation models to map salinity and identify optimal methods for salt detection. It further employs advanced data processing techniques to refine land cover classification and identify unique features within four sabkhas along the Red Sea coast: Ras Baghdadi, Marsa Abu Madd, Bir Shalatein-Marsa Himeira, and Diib. Examining these sabkhas reveals intricate details of their topography, hydrology, and sediment composition. The study identifies factors contributing to their individual characteristics, such as structural control, interaction with lagoons, and the influence of wind and aridity. Analysis of satellite data and field observations unveils the presence of salt ponds, dunes, microbial mats, and distinct sediment layers within these formations. Evaporite crystals, halophytic vegetation, and color patterns provide further insights into their formation processes. The study emphasizes that sea level fluctuations, fluvial and aeolian processes, and limited human intervention have shaped the temporal evolution of these sabkhas. However, climate change poses significant future challenges. By highlighting the importance of understanding and preserving these ecologically and economically valuable ecosystems, this research underscores the urgent need for their protection in the face of a changing climate.

**Graphical Abstract:**

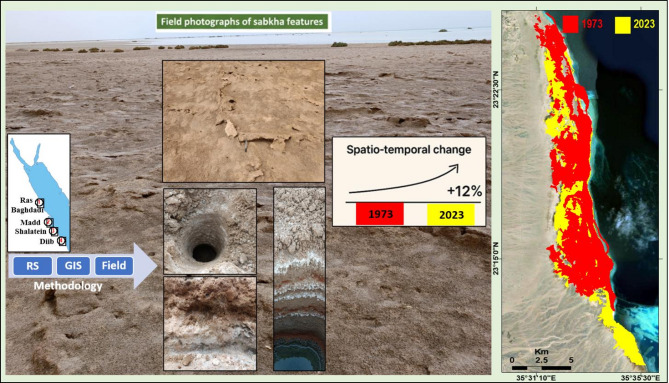

## Introduction

Sabkhas, or salt flats, are diagnostic landforms of arid and semi-arid coasts where intense evaporation precipitates evaporites in closed depressions^1^. Their development proceeds through marine inundation, regressive sediment mixing, and final desiccation under extreme heat and wind, producing saline, crust-veneered plains^2^. Geologically, sabkhas record syn-depositional growth of evaporites within flat-lying terrains; bed thickness is governed by the height of capillary rise above the water Table^3^. Brine chemistry and salt mineralogy reflect the interplay of climate, topography, and antecedent geology^4^.

Hydrologically, sabkhas divide into coastal and inland categories. In coastal systems the water body remains hydraulically connected to the sea; recharge is dominated by direct rainfall and upward groundwater seepage, whereas lateral runoff is negligible^3^. The flats occupy topographic lows whose water table lies within the capillary fringe, typically few meters below the surface depending on pore size^5^. Solutes are supplied by precipitation, storm runoff from adjacent highs, and leakage from the marine water table. Capillary wicking transports brine to the surface where evaporation exceeds recharge, triggering precipitation of calcite, dolomite, gypsum, anhydrite, and halite as surficial crusts and interstitial cements^3^.

Since the first modern descriptions along the Arabian Gulf^6^, comparable settings have been recognized worldwide. Egyptian coastal sabkhas fringe the Gulf of Suez, the Red Sea, and the Mediterranean^7,8^. The Abu Rudeis-Ras Muhammad-Nabq sector forms a well-defined chain, whereas the Bardawil lagoon margin hosts both beach-dune and fluvial variants. West of Bardawil, an 8-km-long, several-hundred-meter-wide depression behind an oolitic ridge at El-Hammam constitutes a classic Mediterranean sabkha^9,10^. Along the Egyptian Red Sea coast, Pleistocene embayment salinas (khors) were isolated by falling sea level and transformed into evaporitic drawdown basins^11^. At Ras Shukeir, a Late Pleistocene sabkha formerly filled with laminated subaqueous gypsum is now an eroded sabkha; laminated aragonite may represent the Holocene analogue^12^.

Previous work has concentrated on sedimentological and mineralogical zonation, commonly portraying concentric suites of carbonate, gypsum, and chloride salts^9,11,12^. However, these studies have left several critical gaps and limitations. First, surface patterns have rarely been linked explicitly to first-order geological controls such as structural lineaments, topographic confinement, or fluvial inputs. Second, the southern Red Sea coast is markedly less documented than the Gulf of Suez or northern Sinai, and its distinct tectono-morphological setting implies a different sabkha style. Third, although remote sensing has been applied sporadically, a systematic, high-resolution workflow integrating multi-temporal satellite imagery, digital elevation models, and targeted field validation is lacking for this region. Our work directly addresses these gaps by not only describing the sabkhas but by deciphering the underlying processes that control their formation and evolution.

This study addresses these deficiencies by coupling detailed surficial mapping with genetic analysis of coastal sabkhas along the southern Egyptian Red Sea. The primary aim is to characterise their spatial distribution and to identify the geological and environmental factors that govern their formation and evolution. Specific objectives are:

1. Generate high-resolution spatial maps of selected sabkhas by fusing Sentinel-2 A imagery with GIS tools to delineate extent and internal facies.
2. Quantify the influence of structural controls (faults, fractures), topography, and sea-level fluctuations on sabkha morphology.
3. Evaluate the role of surface drainage networks in modulating water and solute budgets.
4. Synthesis these datasets into a conceptual model of sabkha spatio-temporal evolution and assess vulnerability to future environmental change.

The resultant workflow and process-based model are transferable to other arid littoral zones and provide a geospatial baseline for resource evaluation, coastal management, and protectorate zoning. Limitations stem from the inherent resolution of satellite data and the logistical difficulty of comprehensive ground truthing in a soft-sediment coastal environment; nevertheless, the approach offers a robust, cost-effective foundation for sustainable land-use planning along the southern Red Sea.

## Study area

**Fig. 1:**
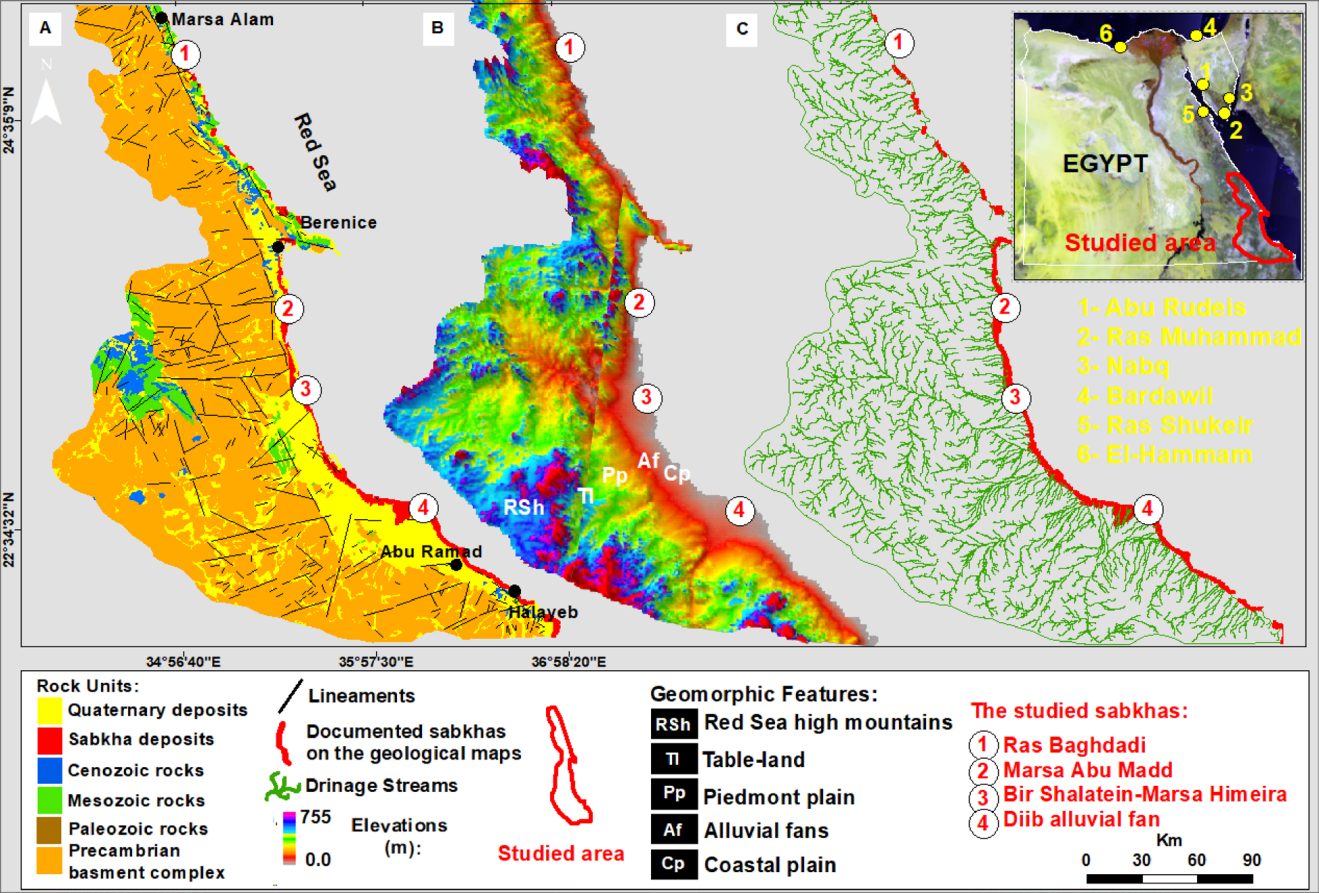
Landsat image of the study area along the Red Sea showing: (A) Location and geological map modified after^13^; (B) Topographic map from Landsat and DEM data; (C) Surface stream networks.

**Fig. 2:**
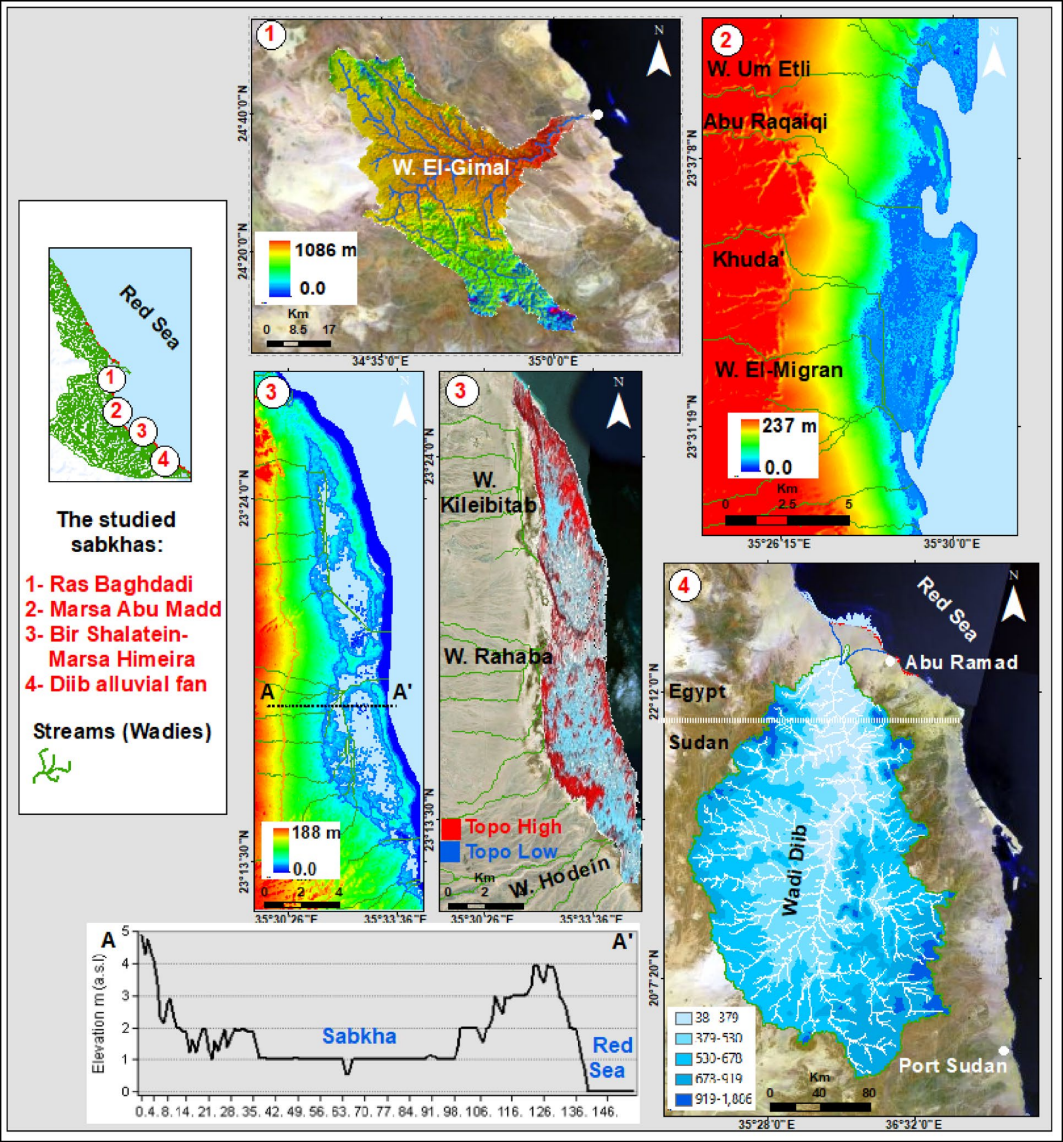
Topographic and geomorphological characterization of the four studied sabkhas along the southern Red Sea coast of Egypt, derived from Landsat imagery and DEM data.

Along a 364-km sector of the southern Red Sea coast, multispectral remote sensing resolved four discrete sabkha bodies, each distinguished by unique spectral signatures and surface characteristics. The study area is located between latitudes 25°04′12.0′′N and 22°26′29.9′′N and extends from Marsa Alam to Abu Ramad, as shown in (Fig. 1). Over the past two decades, the study area has undergone substantial coastal transformation driven by tourism, urban, and harbor development, compounded by natural factors. The once pristine shoreline has been replaced by a built-up coast dominated by hotels and recreational infrastructure. One of the studied sabkhas, known as Ras Baghdadi, is situated 50 km southeast of Marsa Alam and covers an area of around 5.5 km^2^ (Fig. 2-1). The sabkha surface sustains a subtle topographic gradient, with elevations constrained between 5 m and 1.5 m a.s.l. It exhibits an inverted deltoid shape, with its downstream end extending towards Wadi El-Gimal and forming an arrowhead shape (Fig. 2-1). The ecological significance and uniqueness of Wadi El-Gimal have led to the designation of this area as one of Egypt’s national protectorate zones.

**Fig. 3:**
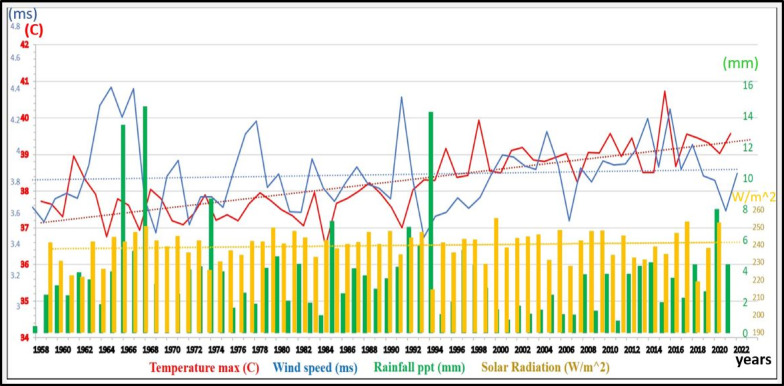
Climatic data showing various parameters from 1958 to 2023 (obtained from^31,32^.

**Fig. 4:**
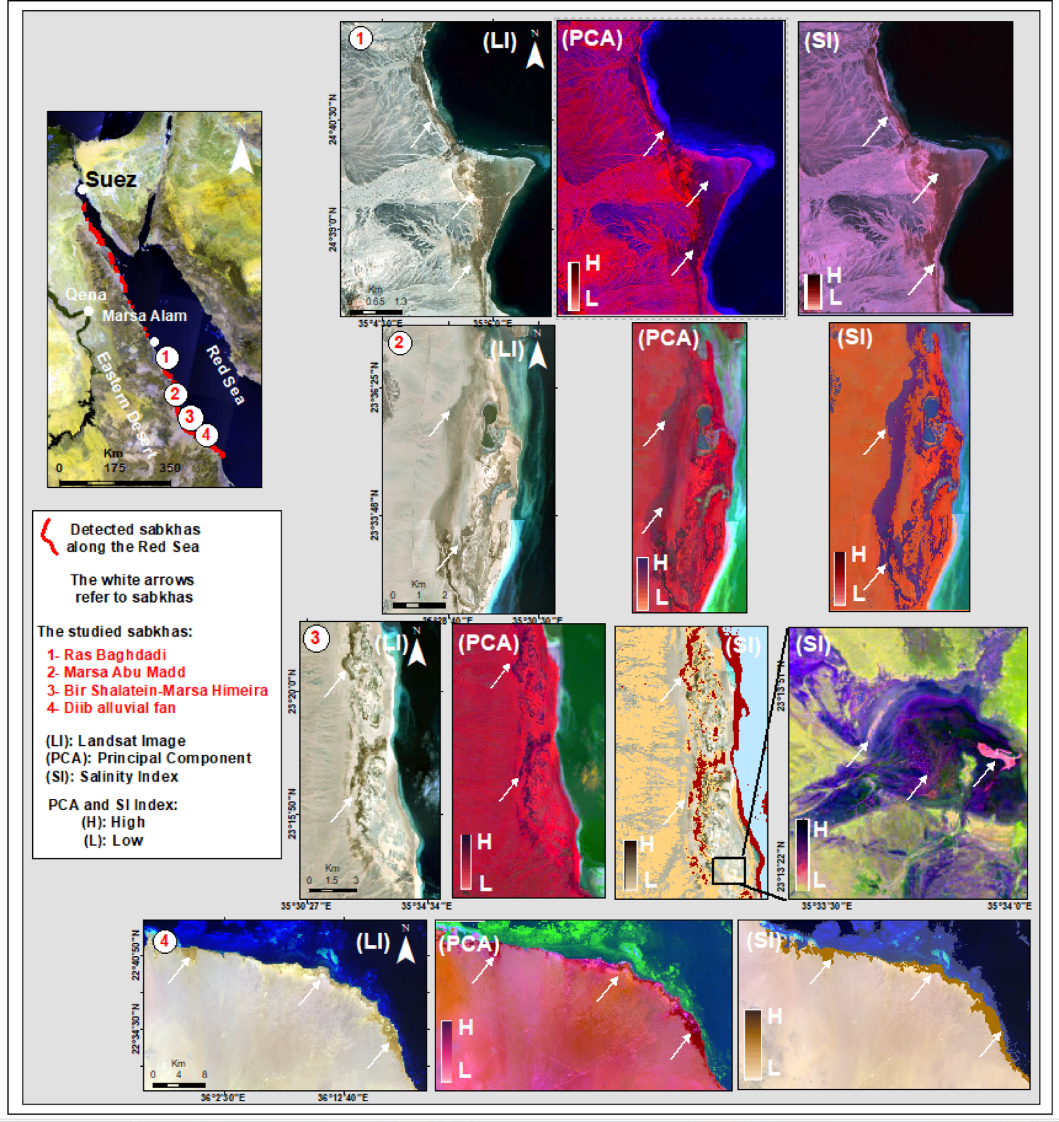
Landsat images showing locations, Salinity Index (SI), and Principal Component Analysis (PCA) of four sabkhas: (1) Ras Baghdadi; (2) Marsa Abu Madd; (3) Bir Shalatein-Marsa Himeira; and (4) Diib alluvial fan.

Marsa Abu Madd, situated 60 km southeast of Berenice town, is a sabkha that stretches approximately 24.1 km parallel to the Red Sea coastline. With an average width of 3.41 km, it takes on an oval shape, encompassing a total area of about 62.7 km^2^ (Fig. 2-2). Notably, this sabkha features a distinct swamp and lagoon in its northern region. The elevations range from 3 to 5 m a.s.l, and several small wadis, including Wadi Um Etli, Abu Raqaiqi, Khuda’, and Wadi El-Migran, drain eastwards towards the plain. Located 70 km northwest of Shalatein, Bir Shalatein-Marsa Himeira there is a sabkha that spans approximately 76 km^2^. Its oval shape measures 22.6 km in length and 3.5 km in width (Figs. 2 and 3), and it boasts relatively high elevations ranging from 4 to 7 m a.s.l. This sabkha is characterized by a high-elevated area as a coastal scarp, which is drained from the east by three major alluvial fans of Wadi Hodein in its southern part, Rahaba in its middle section, and Wadi Kileibitab in the north (Figs. 2 and 3). These fans flow from the high basement mountainous areas in the west. The sabkha, known as Diib Alluvial Fan is situated in the region between Shalatein and Abu Ramad. Its shape resembles that of a delta or fan, with a strip extending 51.9 km along the sea shoreline and 1.12 km towards the southwest, covering an area of approximately 80 km^2^. The elevation of this sabkha ranges from 2 to 5 m a.s.l near the coast, as depicted in Figs. 2, 3 and 4. It is located on the shoreline of the downstream triangular area of a vast drainage system originating from central Sudan.

## Materials and methods

In this study, remote sensing data was extensively utilized to monitor salinization, with a strong focus on accurate soil salinity mapping. The mapping methods played a crucial role in achieving this objective. One of the fundamental methodologies employed in this study is based on change detection, which involves a thorough examination of satellite images from 1973 to 2023 to assess the spatio-temporal evolution of the sabkhas. The multispectral satellite imagery, specifically the Sentinel-2 A data, emerged as a prominent candidate for salinity monitoring, which has been the subject of extensive investigation in recent years^14^. Equipped with a state-of-the-art multispectral instrument, the Sentinel-2 satellite provides high-resolution optical images. The data employed in this research were acquired by the Sentinel-2 A optical imaging satellite in May 2023. The satellite’s multispectral instrument comprises 13 spectral bands, four of which have image bands with a spatial resolution of 10 m (B2, B3, B4, and B8), six image bands (B5, B6, B7, B8a, B11, and B12) with a spatial resolution of 20 m, and three image bands (B1, B9, and B10) with a spatial resolution of 60 m. A diverse range of the electromagnetic spectrum, comprising visible, NIR (red-edge bands), short-wave infrared, and four red-edge bands, are covered by these spectral bands, rendering them exceptionally useful for salinity monitoring. Furthermore, the ALOS PALSAR digital elevation model (DEM) with a resolution of 12.5 m was procured from JAXA, and it was subsequently processed by the Alaska Satellite Facility^15^. This DEM data enabled us to deduce crucial topographic characteristics and surface stream networks that are pertinent to the spatial models of salt flats.

Radiometric and geometric correction projected to the Universal Transverse Mercator (UTM) and World Geodetic System 1984 (WGS 84). The Sentinel-2 A imagery, DEM, climatic data, and geologic map were then processed using ArcMap 10.8.4 software, in the creation of detailed salinity maps. These maps facilitated the identification of sabkha spatial distributions.

Additionally, the field work served to establish ground-truth essential for validating the remote sensing data. This involved using a GPS unit to accurately map the boundaries and extent of the sabkha areas. Detailed direct observations were conducted to characterize the surface features, including the type of salt crusts and underlying sediments. Crucially, soil/sediment samples were collected at representative sites for lab analysis of salinity, providing the necessary quantitative data to calibrate the remotely-sensed Salinity Index (SI). Recently, several salinity indicators have been developed for detecting areas of soil affected by salt using satellite imagery^16^. These indicators are mainly based on the spectral signature of saline soils in different bands of satellite sensors. Referred to as the Salinity Index (SI), they effectively emphasize the spectral reflectance of salt crusts on the soil surface. It is worth noting that many studies have employed multi-temporal remote sensing data across a broad range of the electromagnetic spectrum to compute soil salinity indicators^17^.

A comprehensive analysis was conducted in the present investigation on eight distinct spectral salinity indices that were previously developed in various research studies. These indices, namely SI 1, SI 2, SI 3, SI 4, SI 11, NDSI, VSSI, and NSI, were proposed by^18–22^. After careful assessment, the salinity indices that demonstrated the most appropriate and optimal performance were identified as NDSI which can be computed as (band11-band12)/(band11 + band12), B/R ratio, and the Sqrt Green multiplied by Red, as described by^18,21^. These selected indices consistently yielded logical and accurate outcomes, aligning with the ground-truth data obtained in this research. To optimize the processing and visualization of the data, Principal Component Analysis (PCA) was implemented in the present research. PCA, a robust statistical technique for enhancing images, is widely employed in the fields of pattern recognition and spectral transformation to effectively reduce data redundancy^23^. By representing the data using the first two or three components, PCA enables the visualization of maximum spectral contrast through the utilization of three primary display colors. The efficacy of PCA in assessing salinity has been demonstrated by^24^ in their investigation of salt-affected soil in arid regions. In the present study, PCA was specifically applied to the salinity indices derived from Sentinel-2 A satellite imagery data, resulting in the extraction of the main components.

For the supervised classification of Sentinel-2 A satellite image data, the Maximum Likelihood Classification (MLC) method was employed, which is widely utilized for the analysis of satellite imagery. MLC involves the utilization of a discriminate function to assign pixels to the class with the highest likelihood^25^ results of each pixel against the definitive land cover conditions obtained from corresponding ground-truth data.

This approach provided a reliable measure of the classification’s accuracy, calculated as the percentage of correctly classified validation pixels out of the total number of pixels in all the ground-truth classes^26^.

## Geology

The Red Sea Mountains form a linear coastal chain of Precambrian massifs flanked by younger sedimentary veneers. Crystalline basement composed of Precambrian rocks succession from psammitic gneiss (oldest) through hornblende gneiss, schist, dismembered ophiolitic mafic-ultramafic, metavolcanics, granodiorite, biotite granite to late muscovite-plagioclase granite and quartz veins^27,28^ (Fig. 1A, B). These rocks are onlapped eastward by Mesozoic strata, Miocene evaporites, and Pliocene-Pleistocene clastic and marine deposits that record rift-flank uplift and episodic marine incursion; Pliocene beds were laid down close to the present shoreline as uplift progressed^29^. Quaternary sand sheets, dunes and alluvial fans prograde east-west down wadi systems, while fringing reefs build the littoral plain seaward^29,30^ (Fig. 1A, B). The variations in lithology and microfacies within the coastal plain’s sedimentary record document a history of shifting depositional environments from continental terrestrial and fluvial settings to open, shallow marine intertidal, supratidal, and lagoonal conditions. These environmental shifts, recorded over time, are associated with changes in climate from hot-dry to warm-wet. The Basement Complex and the entire overlying sedimentary succession within the study area exhibit various geologic structures, including faults, with the eastern edge of the basement being fault-controlled in some areas and unconformity-demarcated in others (Fig. 1A). The drainage and deeply incised wadis are structurally controlled, and the coastal plain is fault-modified. The main structural lineaments in the area indicate predominant trends in the N45W, N25E, N5W and N45E, N5E, E-W, N25W, and N55W directions.

## Geomorphology

The study area exhibits five prominent geomorphic features, namely the Red Sea high mountains, the table-land hills, the Piedmont plain, the alluvial fans, and the coastal plain (Fig. 1B). Among these features, the Red Sea Mountains stand out as the most significant, made up of Precambrian igneous and metamorphic rocks. Deep valleys dissect the mountains, and their summits are often capped by plateaus. Adjacent to the high mountains of the Red Sea are the table-land hills, which are composed of metasedimentary and metavolcanic rocks of Precambrian age [e.g., 27, 28]. These hills are generally lower in elevation compared to the mountains with rounded summits.

East of the high-mountain block and table-land hills, a broad piedmont plain of fluvial sand, gravel and clay descends toward the coast. At the mouths of wadis, coarse-grained alluvial fans boulders to clay prograde basin ward; their distal fringes are overlain by vegetated coastal sabkhas. The narrow coastal plain, bordered by rift shoulders and fault scarps, incorporates raised beaches, eolian dunes, sabkhas and mangroves. A modern fringing reef extends 50–100 m offshore but is locally extinguished at wadi mouths by flood-borne sediment; the adjacent beach lies 2–1 m a.s.l.

The elongate Precambrian block forms the Nile-Red Sea watershed; sub-parallel wadis, aligned to the eastward regional slope, incise the range and discharge into coastal embayments that trap all surface runoff (Fig. 1C). These embayments sculpt the shoreline and modulate near-shore marine ecosystem health.

## Climate and meteorology

The study area lies in an arid to semi-arid belt of high insolation, strong winds and extreme evaporation^31,32^. Climatic records (1958–2023) yield a mean annual rainfall of 6.5 mm, delivered as short, high-intensity winter showers; summer temperatures peak at 37–40 °C, winter minima range 8–22 °C, and relative humidity oscillates between 27% (summer) and 58% (winter) (Fig. 3). Evapotranspiration intensifies from 8.5 mm/day in winter to 28 mm/day in summer under a solar flux of 234–256 Wm⁻². The Marsa Alam-southern sector receives ~ 98.8 × 10⁶ m³ yr⁻¹ (~ 80% of the total), This influx of meteoric inputs provides a significant source of weathering products and minerals, which can replenish the adjacent sabkha ecosystems^33^.

The coast is affected by persistent NW-N-NE winds averaging 4.2 m/s; diurnal land-sea heating intensifies daytime speeds, driving sandstorms and erosion. Wind-forced waves adopt a NE-SW alignment, enhancing breaker energy and longshore drift. Tidal patterns in the Red Sea follow a semidiurnal rhythm, reaching their peak every 12 h. It is worth noting that there is a difference in tide levels between winter and summer, with winter tides surpassing summer tides by 0.5 m. On average, the tidal range measures approximately 0.65 m, but it can reach up to 0.95 m^34^. It is worth mention that, the salinity signature of the Red Sea in the study area is characterized by an elevated salinity range of 38.5 to 39.5 parts per thousand (ppt), primarily attributed to the substantial evaporation coupled with a low mean annual precipitation regime^35^.

## Results

### Remote sensing and spatial mapping

The application of salinity indices (SI’s) effectively distinguished the sabkha regions from the surrounding sedimentary environments. Graphical representations of these outcomes are illustrated in Fig. 4 (SI 1 to 4), with a graduated rainbow color scheme employed to represent values ranging from light violet (lowest) to dark red (highest). Light gray color hues indicated the presence of fine sediments, while darker shades of gray were indicative of higher water content, a shallower water table, and higher clay content. The Wadi drainages appeared as shades of light gray, while the sabkha zones exhibited a range of light to dark red, brown, and blue hues, with lighter shades representing arid conditions and deeper shades indicating wetter areas. The imagery effectively captured the distinct attributes of sabkha and its surroundings (Fig. 4 LI & SI 1 to 4).

Significantly, the light gray color hues were used to indicate the presence of fine sediments. In contrast, darker shades of gray were indicative of higher water content and a shallower water table, along with high clay content. The Wadi drainages appeared as shades of light gray, while the sabkha zones exhibited a range of light to dark red, brown, and blue hues. The nuances of the dark red, brown, and blue hues corresponded to dry and wet conditions, with lighter shades representing arid conditions and deeper shades indicating wetter areas. The presence of loess sediments was skillfully portrayed in gray and fuchsia hues (Fig. 4 LI & SI 1 to 4). The tonal variations observed within the sabkha areas can be attributed to the dynamic interplay of multiple factors, including sediment composition, mineralogy, moisture content, groundwater salinity, and the depth of the water table.

Principal Component Analysis (PCA) resulted in enhanced images that provided valuable insights, with sabkha features prominently depicted using PC7, PC5, and PC3 in the red, green, and blue channels, respectively (Fig. 4 PCA 1 to 4). A supervised classification was performed for each sabkha site to refine the current understanding, drawing on an extensive ground-based investigation of 150 checkpoints. The primary goal of the Land Use and Land Cover (LU/LC) analysis was to clearly differentiate and separate the sabkha areas from the surrounding rock types. The resulting boundaries, illustrated in Fig. 4 (PCA 1 to 4), were subsequently validated through rigorous field verification. Critically, the absence of any observed human activities within the sabkha regions confirms their pristine and undisturbed nature.

### General Sabkha characteristics

A comprehensive analysis of 330 Sentinel-2 A Landsat images was conducted to reveal the spatial distributions of sabkhas along the southern coast of the Red Sea. Through the integration of Landsat imagery, topographic data, and geological maps, along with on-site field investigations, a total of four distinct coastal sabkhas have been identified in a north-to-south sequence: Ras Baghdadi, which exhibits structural control; Marsa Abu Madd, which is linked with adjacent lagoons; Bir Shalatein-Marsa Himeira, primarily influenced by aridity and topography; and Diib Delta, representing a fluvial-deltaic sabkha at the shoreline (Figs. 1 and 2). The sabkhas under investigation are situated within oblong to elongated depressions, frequently arranged in parallel with the coastline of the Red Sea, and are interconnected with the downstream areas of wadis (Fig. 2). These depressions exhibit a continuous hydraulic connection with the sea, facilitated by relatively narrow incisions that traverse the Late Pleistocene reef-and-beach barrier. Consequently, these incisions allow for the accumulation of sedimentary deposits originating from both sabkha and alluvial sources. Sabkha surface conditions ranging from inundated to desiccated are highly sensitive to the combined effects of seasonal water table variations, tidal action, and the balance between precipitation and evaporation.

**Fig. 5:**
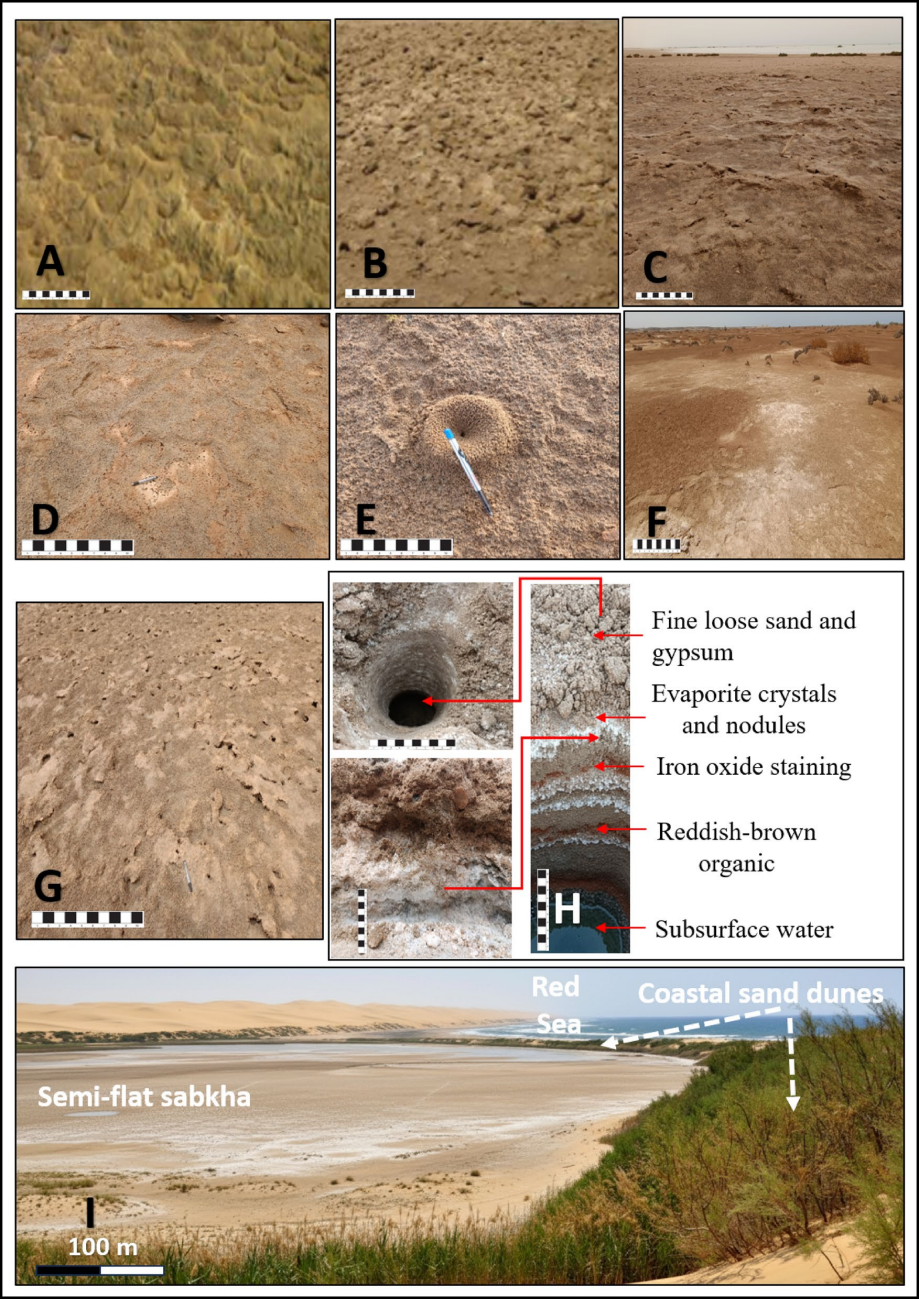
Field photographs of sabkha features (Scale bar = 10 cm): (A) Adhesive ripples; (B) Embryonic teepee polygonal; (C) Mature tepee; (D) Extensive salt crust; (E) Gas bubbles/blisters; (F) Conical and flat-topped surface with halophytes (mixed sandflat/mudflat); (G) Wrinkle structure; (H) 50 cm deep pit with fine sand layers disturbed by gypsum growth; (I) Semi-flat sabkha separated from the sea by coastal sand dunes.

Based on our field observations, we identified several surface features that are common to all four studied sabkhas. In wet sabkha sediments, we observed surface features such as adhesive ripples (Fig. 5A), and embryonic tepee polygonal formations (Fig. 5B). In contrast, the drier sabkha areas revealed mature tepee structures (Fig. 5C) and extensive salt crusts (Fig. 5D). Gas bubbles and blisters (Fig. 5E) were also noted. In the transitional zones between the wetter mudflats and drier sandflats, we frequently observed conical and flat-topped micro-topographical features stabilized by halophytic vegetation (Fig. 5F). These mixed sandflat/mudflat surfaces are colonized by salt-tolerant plants, whose roots help bind the sediment and create small, elevated mounds. Additionally, the fine, sinuous patterns known as wrinkle structures (Fig. 5G) were commonly observed on the surfaces of cohesive microbial mats. These features were consistently documented across the Ras Baghdadi, Marsa Abu Madd, Bir Shalatein-Marsa Himeira, and Diib sabkhas.

A pit dug to a depth of 50 cm unveiled of the sabkha alternating strata of tidal and windblown sediments. These layers were primarily sandy and exhibited distinct dry evaporite banding (Fig. 5H). The uppermost 10–20 cm of these bands were altered by the presence of evaporite crystals and nodules, while the surface layer displayed porous, loose sediment with a vuggy texture. Below the surface layer, sporadic red staining stemming from iron oxides and hydroxides was observed in the wet sediment beneath (Fig. 5H).

The studied sabkhas are characterized by their relatively semi-flat topography and are typically arid. They are geographically separated from the sea by coastal sand dunes (Fig. 5I). Compared to both coastal dunes and sabkhas, beach sediments are generally coarser. As sediment is transported inland, the sorting and grading of grain size improve, resulting in a relative increase in the finer fractions of coastal sand dune sediments. The salt-flat zones are surrounded by mudflats that are saturated with brine and are home to sporadic halophytes. The groundwater level beneath sabkhas is measured to be between 50 and 75 cm below the surface (Fig. 5H). This level can fluctuate when both marine and non-marine waters infiltrate the salt flats, particularly when the groundwater table is close to the surface of the sabkha deposits. The size of the salt flats can vary depending on factors such as the amount of groundwater seepage, the slope of the terrain, and the surface area of the evaporite depression. These salt flats can range from a few square meters to hundreds of square meters in size. During the summer, the surfaces of sabkhas are covered with layered salt, while in the winter, they are submerged in water.

### Individual Sabkha descriptions

#### Ras Baghdadi Sabkha

**Fig. 6:**
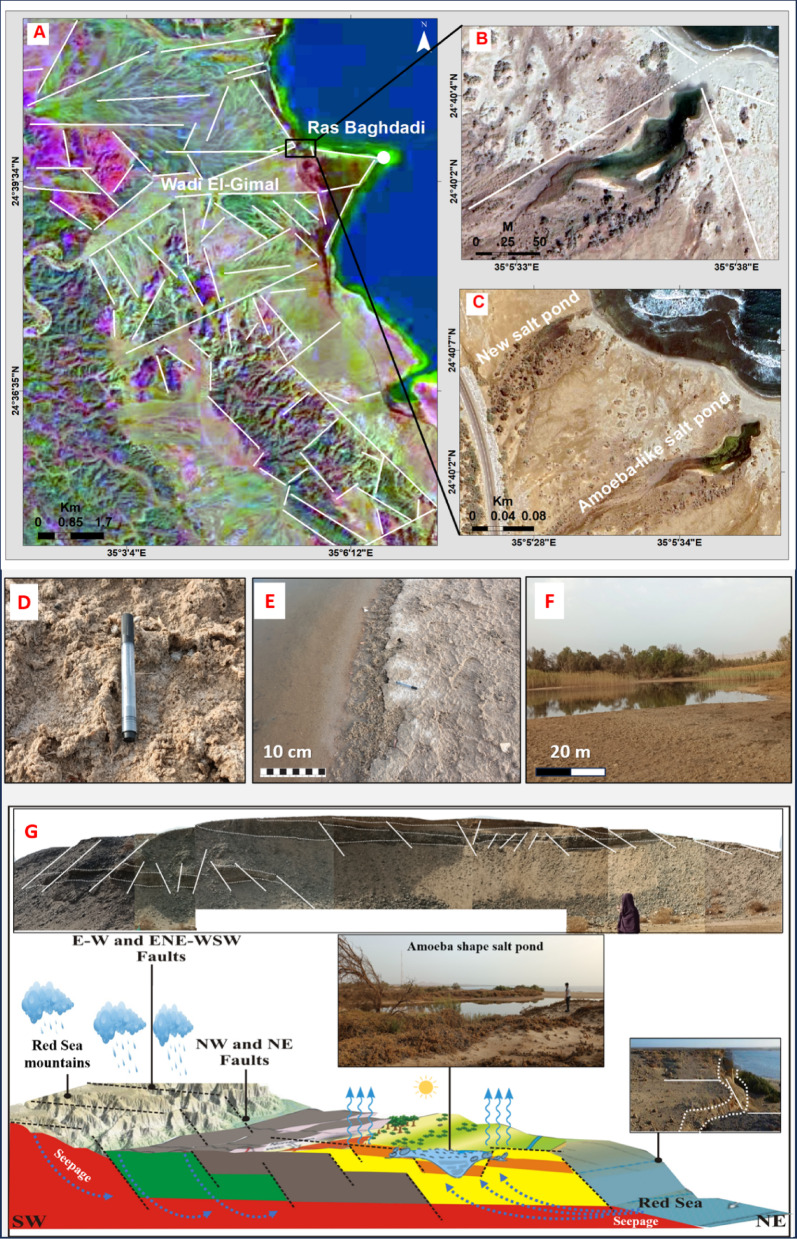
Coastal and Structural Dynamics of Ras Baghdadi Sabkha; (A) Structural lineaments controlled sabkha; (B) An amoeba-like salt pond and identified lineaments (white lines), alongside the Wadi El-Gimal drainage pattern; (C) Embryonic new pond; (D) Salt crystal and microbial mats; (F) Salt microbial mats transition to tepee at the pond periphery; (G) Hypothetical 2D structural model of Ras Baghdadi sabkha.

This particular sabkha occupies a remarkably well-structured alluvial fan that is under the influence of sets of major normal faults, running in an ENE-WSW, ESE-WNW, E-W, and an NW-SE direction (Fig. 6A). These faults originally controlled the drainage of Wadi El-Gimal (Figs. 2 and 6A-1). Additionally, there are a series of faults on the cliffs, including a significant NW-SE trending fault that limits the basement complex to the east and affects the Quaternary and reef platforms. Notably, one of these fault blocks, located approximately 1.3 km from the shoreline, is partially covered by alluvial gravel and can be clearly seen in the channel running through the main cone. The second fault, less than 1 km from the coast, forms an escarpment that connects to a narrow platform with coastal cliffs on the eastern side. The fan is also affected by a distinct set of NE-SW faults, which can be observed on the salt-crusted surface through irregular elongate depressions and mounds (Fig. 6A). Along the main ENE-WSW and ESE-WNW faults that cut through the northern part of the sabkha, there is an isolated salt pond with a shape resembling that of an amoeba (Fig. 6B & C).

This fault zone allows water to flow from both the surface drainage on the west and the seepage of seawater from the east through the permeable fluvial sands; generally, these sabkha are structurally controlled (Fig. 6A & G). Consequently, a stagnant pool of highly saline water is formed due to the high rate of evaporation. The water level in the pond is relatively shallow, ranging from 50 to 75 centimeters, and is slightly lower than the surrounding sediment surface. The pond is surrounded by elevated mudflats saturated with brine, interspersed with scattered halophytes (Fig. 6F). The water table in this area is near the surface of the seawater (Fig. 6E & F).

The observed zonation within the pond from algae and seagrass in the slightly deeper central waters to white evaporite crusts at the margins was noted (Fig. 6E). The sandy sediment within the pond contains distinct salt crystals and layered microbial mats that can reach a thickness of 10 to 20 centimeters (Fig. 6D). These mats transition laterally into buckled tepee structures at the periphery of the salt pond (Fig. 6E). Additionally, embryonic ponds connected laterally to the sea have also been observed. One such pond is located in a topographic depression further north, parallel to the main pond (Fig. 6C).

#### Marsa Abu Madd Sabkha

**Fig. 7:**
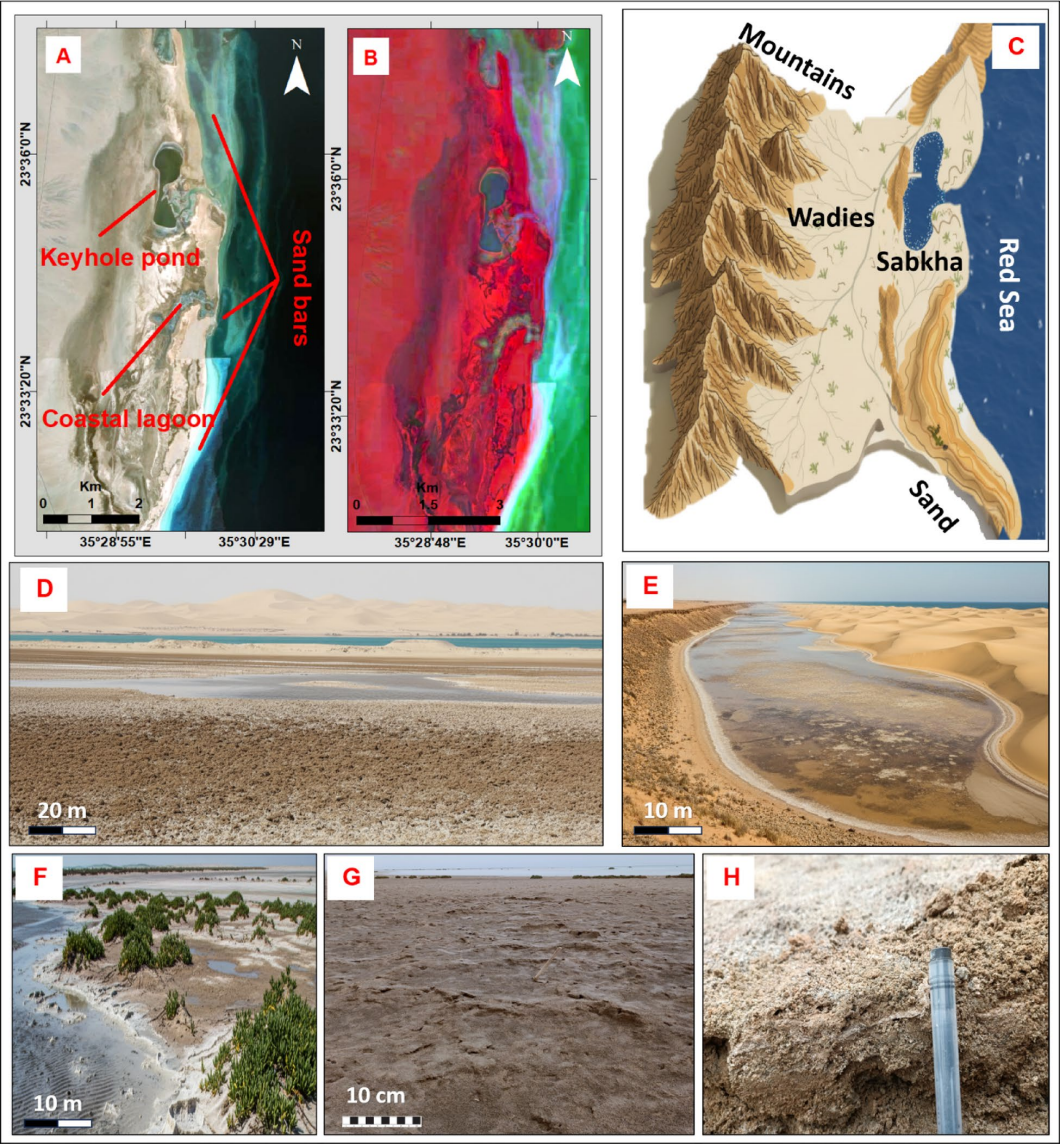
Aspects of Marsa Abu Madd sabkha: (A) Image showing its irregular oval shape, open semi-isolated lagoons, and the keyhole pond; (B) Interpretive map, of sabkha plain show dark gray with violet reddish hues of fine-grained sediments; (C) Hypothetical model; (D, E) Sand bar and lagoon with shallow sea-water; (F) Fine-grained sediments with dark gray and violet reddish hues; (G) Polygonal and tepee formations; (H) Salt and evaporite crystals.

This sabkha displays an irregular oval shape and appears as a flat expanse that is low-lying, with arid highlands bordering its western periphery and the Red Sea forming its eastern boundary (Fig. 7A, B, C). The sabkha plain is bounded and separated from the adjacent lagoon by a series of coastal sand bars. These barriers, formed by the reworking of coastal sediments by waves and currents, play a critical role in limiting direct hydraulic exchange and creating the isolated environment necessary for sabkha development (Fig. 7C–E) The eastern region of the sabkha transitions into coastal lagoons, with clear demarcations indicating the presence of an expansive open lagoon in the northern sector. This lagoon is characterized by shallow water depths ranging from 0.75 to 1 m (Fig. 7D, E). Additionally, there is an isolated pond with a distinctive keyhole configuration, along with semi-isolated lagoons nestled within the central portions. These lagoons are effectively separated from the sea domain by natural sand bars. Close to the coastal interface, the supratidal flat forms the eastern edge of the sabkha.

This area is distinguished by a chromatic spectrum that leans towards dark gray with violet reddish hues and is predominantly composed of fine-grained sediments (Fig. 7F). On the other hand, the inland fringes display polygonal structures and tepee formations (Fig. 7G). Circular mounds created by insular salt and evaporite crystals can also be found (Fig. 7F, H). These alternating layers of aeolian-deposited desiccated sediments are accentuated by subtle yet discernible pale shades. The intensification of the sabkha is indicated by a noticeable deepening of hues towards darker tones (Fig. 7B, F, G), which signifies a higher concentration of evaporite components.

#### Bir Shalatein-Marsa Himeira Sabkha

**Fig. 8:**
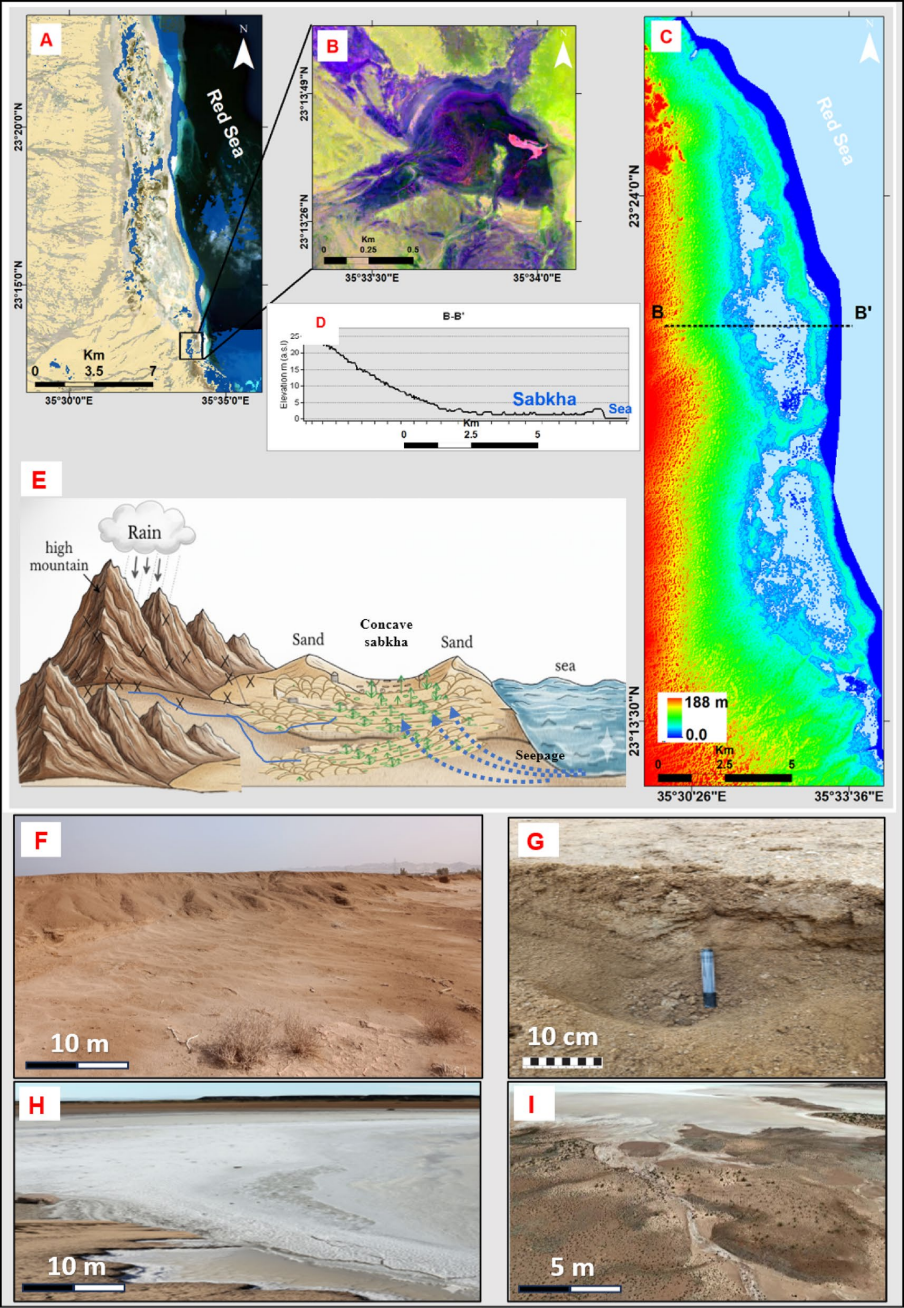
(A) Bir Shalatein-Marsa Himeira sabkha; (B) Crescent-shaped salt flat; (C) Topographic map; (D) Topographic cross section (B-B’); (E) Hypothetical sketch model; (F) Elevated sand, silt and halophytic vegetation on salt flat; (G) Sand and silt with salts; (H) Well-developed sabkha; (I) Hydrological pathways and the entrainment of saline water to sabkha.

This particular sabkha is expanding as a concave depression that occupies lower areas in terms of topography, with elevations ranging from 2 to 4 m (Fig. 8A, B, C, D, E). These depressions are bordered by elevated sand flats, which have elevation gradients spanning from 3 to 7 m (Fig. 8C, D). These sand flats effectively indicate the transition from the arid desert zone. The coastal boundary of the sabkha is determined by a coastal ridge or escarpment, reaching heights of 4 to 6 m a.s.l (Fig. 8C–F). In general, this sabkha is predominantly arid in the interdune sectors and is characterized by the prevalence of halophytic vegetation. On the inland side, its deposits are underlain by layers of sand and silt (Fig. 8F).

Upon careful examination of the southern sectors of the main sabkha, a distinctive and fascinating geomorphological pattern emerges, characterized by an almost circular shape accompanied by a noticeable crescent-shaped extension (Fig. 8B, H). Within this complex, there are observable sinuous channels with a whitish appearance, indicating the presence of internal hydrological pathways and the entrainment of saline water (Fig. 8H, I). The southern part of the main sabkha clearly exhibits a well-developed sabkha environment (Fig. 8H, I), referred to as lacustrine sabkha or plays, according to^36^. This environment is characterized by a nuanced color zoning arrangement (Fig. 8B). The substrate in this context exhibits a dark reddish blue hue, indicative of mud, a light orange violet and brown tint, typifying sandy components, and a resplendent white countenance attributed to salt accumulations (Fig. 8G. H). The eastern extremity of the sabkha presents an intriguing crescent-shaped tail dominated by vivid salt flats, displaying an array of hues, including white, light red, green and orange (Fig. 8B). The white zones stand juxtaposed to the slightly moister dark pink and blue segments. Additionally, intriguing dark circular formations, potentially arising from the presence of algae and organic matter, contribute to the textural and chromatic diversity within the landscape (Fig. 8B)^37^.

#### Diib alluvial fan

**Fig. 9:**
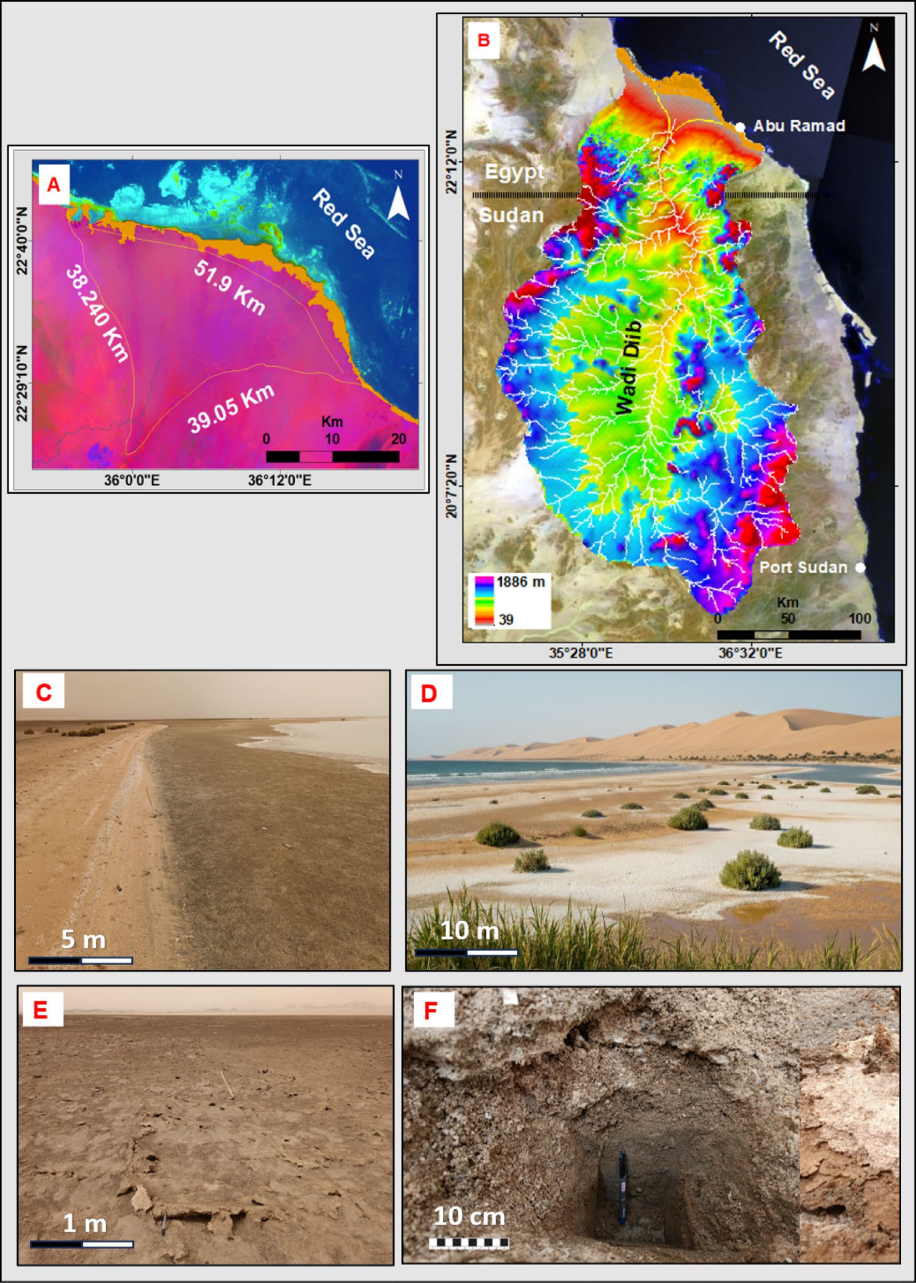
(A) Diib alluvial fan sabkha; (B) Wadi Diib drainage; (C) Seaward sabkha flat; (D) Sabkha and clay plain with halophytes and sand dunes; (E) Well-developed tepee formations; (F) Sand, silty clay and salts.

The sabkha in question is a distinctive irregular stripe that forms along the shoreline of the sea. It is found at the edge of the Diib alluvial fan, which is the most prominent geomorphic feature located at the foothills of the southern Red Sea Mountains in Egypt (Fig. 9A, B). It is worth noting that this sabkha covers the seaward downstream area of Wadi Diib, and it is remarkably flat even when the tides are high and showing well developed tepee formations with mixed sand, silty clay and salts (Fig. 9C–F). The Wadi Dibb Alluvial Fan has a gentle topography and is characterized by extensive cultivation (Fig. 9D). It is also intersected by a network of seasonal drainage channels that resemble a tree-like pattern (Fig. 9B). The Wadi Dibb, which has a triangular shape, is located approximately 31.560 km away from the shoreline, resulting in the formation of a vast alluvial fan (Fig. 9A). This alluvial fan is primarily composed of sand sheets and dunes, along with coarse sand and silty clay. As one moves further away from the coast, the deltaic plain gradually rises in elevation, reaching heights of 2 to 5 m a.s.l in proximity to the coast and 40 to 42 m a.s.l in the more distant areas of the dune field (Fig. 9D).

The Wadi Diib basin is a significant basin that covers a vast area of approximately 42,000 km^2^. Its main stream channel stretches for about 383.730 km, starting from the western part of Port Sudan and ending along the southern coast of the Red Sea in Egypt (Fig. 9B). The catchment zone of this basin consists of elevated terrains, ranging from sea level to 2180 m at the coastal line in the north and at the peripheral water divide of the catchment, respectively (Fig. 9B). On average, the elevation within this basin is around 550 m a.s.l. One crucial aspect to consider is the impact of the Diib delta on the formation of the coastal sabkha.

### Spatio-temporal evolution

**Fig. 10:**
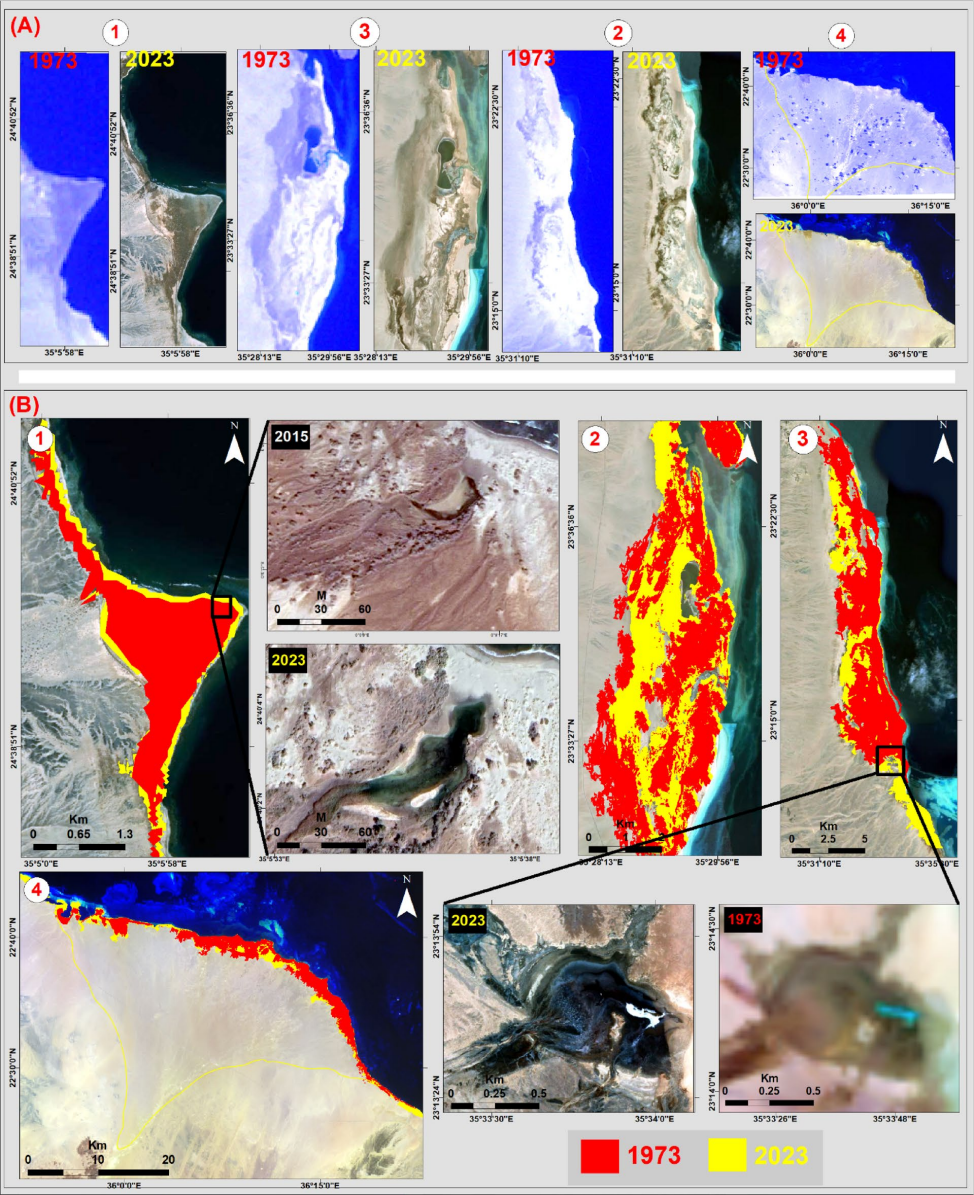
(A): Historical Landsat images 1973 and 2023; (B) Spatiotemporal variations for: (1) Ras Baghdadi; (2) Marsa Abu Madd; (3) Bir Shalatein-Marsa Himeira; (4) Diib alluvial fan sabkhas.

The understanding of spatial-temporal evolution in the context of sabkha formations is of great importance. Through this monitoring framework, a noticeable shift has been identified in the geomorphological characteristics of the studied sabkhas (Figs. 10A-1 to 4 and B1–4). These sabkhas are predominantly confined to the coastline of the research area, with minimal marginal interference inland. A substantial decline in the aggregate expanse of the investigated sabkhas is evident. Specifically, the total extent of the scrutinized sabkhas was approximately 126.38 km^2^ in 1973, which subsequently expanded to 141.71 km^2^ by 2023. This results in a net deviation of 15.33 km^2^, highlighting an expansion in the overall acreage of the coastal sabkha domain (Fig. 10).

The dimensions of Ras Baghdadi sabkha, and it’s an internal lake, underwent a detailed analysis to understand its spatio-temporal evolution. It could be noted that there was a significant increase in the overall extent of the investigated sabkha. Specifically, the total area of the analyzed sabkha was approximately 2.84 km^2^ in 1973, which subsequently expanded to 3.21 km^2^ by 2023, this represents a net deviation of 0.37 km^2^ (Fig. 10B-1). By 2023, the unique internal lake had transformed from its initial crescent shape in 2015 to its present-day amoeba-like structure (Fig. 10B-1). Interestingly, the contiguous headlands, which run parallel to the shoreline, show evidence of gradual progradation and modification over time.

The Marsa Abu Madd sabkha, which covered an area of 19.44 km^2^ in 1973, experienced a significant increase in size, reaching 23.99 km^2^ by 2023, indicating a growth of 4.55 km^2^ (Fig. 10B-2). A notable retreat of the sabkha’s western edge was witnessed in 2023. The centrally located pond has decreased in size, taking on a keyhole shape within the sabkha, while the northward open lagoon has contracted and transformed into a semi-closed lagoon due to the regression of seawater. Over time, this lagoon gradually dries up and merges with the main sabkha area, particularly in concave depressions, receiving sediment from various sources.

In the case of the Bir Shalatein-Marsa Himeira sabkha, its footprint has witnessed a significant expansion from 45.38 km^2^ in 1973 to 47.33 km^2^ in 2023, indicating an increase of 1.95 km^2^ (Fig. 10B-3). This growth is evident through the emergence of sandbars near the shoreline in both the northern and southern parts of the sabkha. The southeastern part of the main sabkha exhibits a prominent characteristic that deserves attention. Throughout the investigation, there has been a noticeable increase in size. Moreover, in 2023, clear zoning characteristics can be observed, indicating the presence of a fully developed salt flat (Fig. 10B-3).

The Diib alluvial fan sabkha, which covered an area of 58.72 km^2^ in 1973, has expanded to 67.18 km^2^ by 2023, resulting in an increment of 8.46 km^2^ (Fig. 10B-4). This expansion, whether towards seaward or landward, can be attributed to the significant influx of sediment caused by the prolonged flow of Wadi Diib during flood events. Additionally, wind-blown effects contribute to the formation of aeolian sabkha deposits during arid seasons, along with the percolation of seawater.

Fortunately, the spatio-temporal analysis reveals that the studied sabkhas are resilient to land use and cover modifications. Anthropogenic impacts resulting from industrial, agricultural, urban, or tourist-related expansions are conspicuously absent in the coastal sabkhas.

## Discussion

### Interpretation of general Sabkha characteristics

The tonal variations observed within the sabkha areas can be attributed to the dynamic interplay of multiple factors, including sediment composition, mineralogy, moisture content, groundwater salinity, and the depth of the water table. The features we identified are indicative of the dynamic interplay between microbial activity, sediment saturation, and evaporite precipitation. For instance, tepee structures (Fig. 5B, C, E) form on the surface due to the enrichment of methanogen bacteria in buried organic matter. The resulting upward diffusion of gas is captured by surface microbial mats, and as salinity increases, salt accumulation causes the mat surface to form domes^38^. The alternating layers of tidal and windblown sediments are the result of the dynamic nature of sabkha ecosystems. Tidal sediments are deposited when the water table rises, while windblown sediments are deposited when the water table falls. The evaporite crystals and nodules emerge when the water evaporates, leaving behind the dissolved minerals. The loose and vuggy surface veneer sediment is due to the high porosity of the sand, enabling efficient water to drain through it. The presence of patchy red staining, attributed to iron oxidation or the presence of organic materials from bacteria and algae, imparts a reddish color to the sediment found in sabkhas (Fig. 5H).

### Controlling factors of individual Sabkhas

The results reveal that while all studied sabkhas are products of an arid climate, their specific geomorphology, sedimentology, and evolution are governed by a unique interplay of local controlling factors. A clear north-to-south trend in dominant influences is apparent.

#### Structural control: the Ras Baghdadi Sabkha

This sabkha stands out as a structurally controlled system, where faulting dictates hydrology, leading to the formation of a unique, isolated salt pond. The intersection of the ENE-WSW and ESE-WNW fault sets has created a localized depression that acts as a focal point for both continental runoff from Wadi El-Gimal on west and seawater seepage from the east (Figs. 2−1 and 6A, B, G). This dual-source input is critical for sabkha formation^3^. The observed zonation within the pond from algae and seagrass in the slightly deeper central waters to white evaporite crusts at the margins is a classic indicator of increasing salinity and desiccation away from the main water body^38^. The layered microbial mats are a key biological component in this environment, trapping sediment and influencing early diagenesis. Their transition into buckled tepee structures at the periphery is a well-documented phenomenon caused by the expansion of evaporite crystals and gas pressure from microbial decay, which deforms the cohesive mats^38^. This observation highlights the complex interplay between structural geology, hydrology, and microbiology in shaping the sabkha landscape. The presence of an embryonic pond further north suggests that these structural controls are creating a series of potential sabkha nuclei, which could evolve into more extensive evaporitic features given sufficient time and continued aridity. This model of fault-controlled sabkha development is consistent with observations from other structurally active arid coastal regions.

#### Marine dominance: the Marsa Abu Madd Sabkha

The Marsa Abu Madd sabkha is a classic example of a lagoon-linked coastal sabkha, where its morphology and hydrology are dominated by its relationship with the marine environment (Fig. 7A, C, D, E). This addresses our study’s objectives concerning topographic effects and surface drainage, but in a marine-influenced context. The series of coastal sand bars act as a critical barrier, restricting direct tidal inundation but still allowing for subsurface seawater seepage (Fig. 7D, E). This creates a unique environment where salinity is moderated by the marine connection, differentiating it from more isolated, arid sabkhas like Bir Shalatein. The presence of the open lagoon, semi-isolated lagoons, and the keyhole pond represents a spectrum of hydrological confinement within the system^3^. The keyhole pond, with its narrow inlet, is likely a feature that will eventually become more isolated from the sea as the sand bar continues to build, potentially evolving into a hypersaline pond or a smaller, embedded sabkha. This system highlights the dynamic interplay between coastal processes (wave and current action building the sand bars) and evaporitic processes on the sabkha plain, a model that is widely recognized in other coastal sabkha environments globally. The phenomenon of deepening hues towards darker tones, which signifies a higher concentration of evaporite components, can be attributed to a combination of factors, including fluctuations in the seawater table and variations in rainfall precipitation.

#### Aridity and topographic control: the Bir Shalatein-Marsa Himeira Sabkha

This sabkha is shaped by its topographic position within a depression, presents a quintessential example of an arid, topographically-influenced coastal plain. Elevated between 4 and 7 m a.s.l, it is less susceptible to frequent marine inundation, resulting in a landscape dominated by continental and aeolian processes. A prominent coastal scarp restricts direct seawater access, while sediment and episodic water are primarily supplied by three major alluvial fans originating from Wadi Hodein, Rahaba, and Kileibitat (Figs. 2, 3 and 8C, D and E). This fluvial input, interacting with high evaporation rates, has produced the distinctive circular and crescent-shaped morphologies in the southern sector, characteristic of a lacustrine or “playa” sabkha setting^36^. The most striking surface expression of the sabkha’s hydrological and chemical gradients is its intricate color zoning, which reflects subtle variations in moisture content, sediment type, and evaporite mineralogy. The landscape is partitioned into a distinct zonation pattern with an approximate 1:2 ratio. White bands, covering roughly one-third of the terrain, represent zones of intense evaporation and salt crust formation. These areas are more exposed to solar insolation and wind, leading to accelerated evaporation and a drier microenvironment. Conversely, the remaining two-thirds of the surface, characterized by dark blue, pink, and orange hues, indicate higher moisture content and significant microbial activity. These zones host stratified microbial mats, including dark circular formations likely composed of algal mats, which contribute to the color palette through the metabolic formation of black iron sulfide coatings on sediment grains^37^ (Fig. 8B, H, I). This zonation also correlates with productivity, with the white evaporative zones being the most productive, while the darker, moister sectors are the least. Consequently, the Bir Shalatein-Marsa Himeira sabkha serves as an excellent modern analogue for interpreting ancient arid-continent sabkha deposits preserved in the geological record.

#### Fluvial-aeolian dominance: the Diib alluvial fan Sabkha

This sabkha represents a distinct fluvial-deltaic category, directly addressing our objective to analyze the impact of surface drainage systems. Its formation and evolution are governed almost entirely by the dynamics of a large transcontinental drainage system originating in central Sudan. Episodic, high-volume flood events from this system transport vast quantities of aeolian and continental sediments, depositing them at the wadi mouth to create a prograding deltaic feature that is subsequently modified by sabkha-forming processes. The low-lying topography (2–5 m) makes the plain highly susceptible to inundation from both fluvial floods and seawater, creating a dynamic environment where continental and marine processes constantly interact (Fig. 9C). This interplay is recorded in the stratigraphy as alternating cycles of aqueous and desiccated sedimentation, providing a vertical record of moist and arid phases. The surface of the Diib sabkha plain reflects its coastal-aeolian character, characterized by a sedimentary mixture of salt compounds and mud accumulating in brownish-hued layers up to one meter thick (Fig. 9C, D). During turbulent storm seasons, the backshore regions experience submersion, while adjacent coastal plains supply sediment for the formation of recent sand dunes that run parallel to the shoreline, shaped by prevailing wind patterns.

This unique sedimentary and hydrological signature is confirmed through satellite analysis. The coastal-aeolian sabkha end member, represented by Diib fan, exhibits a general reduction in reflectance values across all spectral bands, particularly within the shortwave infrared (SWIR) bands (6 and 7). This spectral response is attributed to its high moisture content, both inherent and from periodic seawater incursions, which results in the absorption of a significant portion of incident wavelengths (Fig. 9A).

Consequently, the Diib sabkha serves as a critical modern analogue for ancient deltaic sabkha deposits, which are significant indicators of past shoreline positions and fluvial activity in the geological record. The sheer scale of this system, linked to a drainage basin extending far into the continent, underscores the importance of considering large-scale catchment dynamics when interpreting coastal sabkha evolution.

### Drivers of spatio-temporal evolution

We hypothesize that the significant expansion of the total sabkha area, representing an increase of over 12%, is primarily driven by the combined effects of regional sea-level rise and increased climatic aridity. As global sea levels have risen, the hydraulic connection between the Red Sea and the coastal strata has likely strengthened, elevating the saline water table over a broader area. Concurrently, rising temperatures and altered precipitation patterns in the region, as discussed, would increase evaporation rates. This combination of a higher, more extensive saline water table and enhanced surface evaporation creates ideal conditions for the lateral growth of evaporite deposits, thereby expanding the sabkha margins. While the overall trend is expansion, the specific rate and pattern of growth for each sabkha are likely modulated by their individual geomorphological settings, such as the structural control at Ras Baghdadi or the fluvial inputs at Diib Fan, which either facilitate or restrict this inland encroachment of saline conditions.

The dynamic alterations observed are primarily influenced by various natural processes. These processes include the influences of active fluvial and aeolian sedimentation as well as changes in sea levels due to transgressions and fluctuations. The evolution of coastal sabkhas is closely linked to fluctuations in sea levels and the prevailing arid conditions. The development of these sabkhas occurs during both transgressive and regressive phases, either through the addition of marine sediments due to prevailing currents or through the deposition of aeolian or drifting sands. It is worth noting that the primary drainage systems have a direct impact on the spatio-temporal evolution of all sabkhas, influenced by their sedimentary load and their role in shifting sabkha shorelines through the deposition of new sedimentary layers.

### Implications for management and comparative studies

The findings of this study have direct implications for the sustainable management of the Red Sea coast. The high-resolution maps and understanding of controlling factors generated here provide a robust scientific basis for informed coastal planning. These data can be systematically integrated into management policies in several ways. First, the maps can serve as a baseline for monitoring environmental change, particularly from sea-level rise and increased aridity. Second, identifying structural controls and fluvial inputs allows for targeted risk assessment, informing land-use zoning to prevent encroachment from urbanization. Finally, this scientific foundation supports the development of sustainable eco-tourism and strengthens the case for designating these sensitive areas as protectorates, ensuring their long-term preservation against increasing developmental and climatic pressures.

To evaluate the broader significance of our findings, it is instructive to compare the Red Sea sabkhas with other well-studied systems. Within Egypt, the structurally-controlled morphology of Ras Baghdadi shares comparisons with sabkhas along the Gulf of Suez, where faulting and tectonics play a dominant role in their development^12,39^. However, Marsa Abu Madd significantly forms the lagoon-confined sabkha like of Lake Bardawil in Northern Sinai, which are primarily controlled by the dynamics of the semi-enclosed lagoon and its connection to the Mediterranean^9,11,12^. Moving to the African continent, the arid coastal sabkhas of Mauritania provide useful analogues for understanding the interplay between aeolian processes and marine inundation in shaping these landforms^40^. On a global scale, the classic coastal sabkhas of the Arabian Gulf, particularly those studied in Abu Dhabi, serve as a benchmark. The evaporite zonation we observed at our studied areas are a direct analogue to the systems described from this region, where the interplay between subsurface seawater seepage and arid climate creates classic evaporite sequences^41^.

In contrast, the fluvial-deltaic nature of the Diib alluvial fan sabkha is more comparable to systems in other tectonically active arid regions, such as the alluvial fan sabkhas along the Red Sea in Al Qahmah, south Saudi Arabia, where episodic flood events are the primary driver of sedimentation^42^. The hypersaline evaporite and extensive microbial mats zonation of the Bir Shalatein-Marsa Himeira sabkha show clear parallels with the Chott El Jerid sabkha in Tunisia. This comparison could provide a robust model for developing the Red Sea sabkhas^43^. This comparative approach not only validates our local findings but also provides a proven foundation for regional recommendations.

## Conclusions

This integrated geospatial and field investigation elucidates the formative controls and spatio-temporal evolution of four coastal sabkhas along the southern Red Sea rift margin of Egypt. The synthesis of satellite remote sensing (utilizing Salinity Indices, PCA, and MLC), GIS analysis, and ground-truthing reveals that these sabkhas, while products of a hyper-arid climate, are distinct landforms shaped by discrete geological processes.

Key findings demonstrate that each sabkha represents a specific end-member in a spectrum of coastal evaporitic environments, defined by its primary formative regime:

- Ras Baghdadi is structurally controlled, where ENE-WSW and NW-SE fault systems localize groundwater seepage and form isolated, amoeba-shaped salt ponds.
- Marsa Abu Madd is lagoon-linked, its morphology and hydrology dominated by marine interaction through a system of semi-isolated lagoons and protective sand bars.
- Bir Shalatein-Marsa Himeira is an arid, topographically confined sabkha (playa), characterized by pronounced evaporite and microbial mat zonation driven by continental runoff and intense evaporation.
- Diib Alluvial Fan is fluvial-deltaic, its formation governed by sediment and water discharge from the transcontinental Wadi Diib system, subsequently reworked by coastal-aeolian processes.

Despite these differences in formative regime, consistent across all sites, the sabkha interiors are characterized by extensive salt flats, often bordered by brine-saturated mudflats where halophytic vegetation thrives, a pattern clearly observed in the Marsa Abu Madd and Bir Shalatein sabkhas. A critical factor identified across all sites is the proximity of the groundwater table to the surface, which governs the moisture state of the sabkha, from the dry, cracked crusts of Bir Shalatein to the wetter, ponded areas of Ras Baghdadi. Throughout the annual cycle, the salt flats experience inundation by water during the winter season, transitioning to a substrate characterized by stratified salt layers during the summer months.

Spatio-temporal analysis (1973–2023) documents a net expansion of the collective sabkha area from 126.38 km² to 141.71 km². This ~ 12% growth is interpreted as a response to natural geological and regional environmental drivers, primarily sea-level rise elevating the saline water table and increased climatic aridity intensifying surface evaporation. Importantly, these regions have shown relative resilience to direct human disturbances, as significant anthropogenic impacts have been absent.

This research establishes a process-based framework for sabkha development in active rift-margin settings. The high-resolution maps and evolutionary models produced provide a critical scientific baseline for coastal management, resource evaluation, and assessing the vulnerability of these unique ecosystems to future sea-level rise and climate change.

## Data Availability

The datasets used and/or analysed during the current study available from the corresponding author on reasonable request.
